# Young hands, old books: Drawings by children in a fourteenth-century manuscript, LJS MS. 361

**DOI:** 10.1080/23311983.2016.1196864

**Published:** 2016-06-29

**Authors:** Deborah Ellen Thorpe

**Affiliations:** ^a^ Centre For Medieval Studies, University of York, King’s Manor, Exhibition Square, York, North YorkshireYO1 7EP, UK; ^b^ Transylvania University, USA

**Keywords:** medieval, early modern, drawings, children, child, manuscript, codicology, psychology, interdisciplinary humanities

## Abstract

This article scrutinises three marginal drawings in LJS 361, Kislak Center for Special Collections, Rare Books and Manuscripts, University of Pennsylvania Libraries. It first considers the provenance of the manuscript, questioning how it got into the hands of children. Then, it combines developmental psychology with close examination of the material evidence to develop a list of criteria to attribute the drawings to children. There is consideration of the features that help us estimate the age of the artists, and which indicate that one drawing was a collaborative effort between two children. A potential relationship is identified between the doodles and the subject matter of the text, prompting questions about pre-modern child education and literacy. Finally, the article considers the implications of this finding in both codicology and social history since these marginal illustrations demonstrate that children were active in the material life of medieval books.

## 1. Introduction

Added to manuscripts by scribes or illuminators during the production of a book, medieval marginal illuminations might include and combine defecating monks, tumbling animals, grotesques and various other “weirdnesses” (Lerer, 2009, p. 72). Though the exact intention and meaning of these images is debated, they can seem to reflect a juvenile sense of humour to the modern eye.^1^ Similarly, some marginal “doodles” of human or humanoid figures—scribbled by readers or scribes or used as a method of testing the pen—often have an unsophisticated, childlike quality, with their comically exaggerated and crudely executed features. As Kwakkel (2015) has pointed out, these doodles provided scribes an opportunity to “sidestep seriousness” to finally escape the “narrow horizontal tracks on which the lines of text were written”, and for readers to relieve boredom and help formulate their thoughts.

Though some medieval adult scribes, illuminators, owners and readers responded to manuscripts in ways that we may consider childlike, the relationship between actual children and medieval books is less clear. Lerer (2012) has made an insightful and wide-ranging study of the inscriptions, scribbles and drawings made by literate children in manuscripts, focusing upon Chaucer manuscript Princeton University Library, MS 100. He has reached convincing conclusions about why children inscribed books and about the relationship between early modern children and medieval books, as is explained below. Acker (2003) has examined Columbia University, Plimpton MS 258, a child's primer dating to the late fifteenth century. This manual of religious instruction has its tenets reduced to a minimum, which indicated to Acker that it was intended for elementary education (2003, p. 145). Written in Middle English, it also contains attempts to copy the first commandment, the poor spelling and “awkwardly upright and poorly inked” minims of which indicating that they were the work of a novice hand (2003, p. 147).

Munro’s study (2012) of the works of Cowley (1668) demonstrates that it was not just children’s books that were read by children. In his *Works*, Cowley describes how a book by Edmund Spenser lay in his mother’s parlour, which he “happened to fall upon” (Cowley, 1668, S4v; Munro, p. 62). The young boy found himself “infinitely delighted with the Stories of the Knights and Giants and Monsters, and brave Houses”. It was the influence of this childish encounter with an adult’s book that, according to Cowley, made him a poet “as irremediably as a child is made a Eunuch”. Though this reminiscence may be more literary trope than factual reality (“childishness-real *or imagined*”, Munro, 2012, p. 62; my emphasis), it indicates an expectation that developing children might encounter and read their parents’ books. Aside from these studies, most research has focused upon the relationship between child and text, as opposed to child and material *book*, and most, like Lerer’s and Acker’s research, have concerned older children.

This article scrutinises three marginal drawings in LJS 361, Kislak Center for Special Collections, Rare Books and Manuscripts, University of Pennsylvania Libraries which are catalogued by the library as “crudely drawn figures” (Penn Libraries, n.d.). My analysis first considers the provenance of this fourteenth-century Neapolitan manuscript, questioning how it could pass from the hands of Dominican friars into those of children. Then, it delineates a number of stylistic features of the doodles, which distinguish them from adults’ drawings based upon the principles of developmental psychology. I argue that there is evidence for the age of the artist(s), and explain how differences within one drawing suggest collaboration between two children in different stages of development. I present the findings of an examination of the manuscript in person, which has uncovered material evidence to support the stylistic analysis. In concluding, the article considers the implications of this finding for our understanding of the uses and reuses of the material medieval book.

LSJ 361 is a book of astronomical and astrological tables and Dominican sermons dated to 1327, written in Latin (Black, 2006, pp. 64–65; Kristeller, 1990, p. 638; Penn Libraries, n.d.). A badly damaged inscription in the front pastedown reveals that it was produced in Naples in 1327 by a brother at the Dominican convent in Naples whilst he was a university student (Penn Libraries, n.d.). The contents include tables for calculating the day of the week for any day from 1204 to 1512; commentaries on the gospel and epistle readings for the temporal cycle; and tables and lists for “Biblical, classical, and Mideastern dates” (Penn Libraries, n.d.). Before considering the post-medieval doodles in this manuscript, it is necessary to give more consideration to this early provenance, questioning how it made the journey out of the Neapolitan convent.

## 2. The provenance of LJS 361

In the absence of definitive provenance information, it is not clear exactly how this specialised religious manuscript passed out of the medieval convent into a context in which young children could gain access to it. Research has recreated this journey for English books, with Summit (2008) explaining how books were “transported across time and place, from monastery to well-lighted and guarded modern reading rooms” (p. 2). An important step in this journey was from monastic houses into the collections of post-Reformation households and libraries (Summit, 2008, p. 109). Certain monastic books deemed to be of historic value were “desacralized”, and thus were transformed “from objects of belief into sources for a history of belief” (Summit, 2008, p. 8). The others were destroyed or lost, deemed to be irrelevant by post-medieval collectors. Ker (1941) estimated the scale of the loss, finding that of the 600 books recorded in the medieval catalogue of the Austin friars of York, only 5 survived (pp. xi–xii; Summit, 2008, p. 102).

But how did the survival of medieval monastic books compare in Italy, where there was no Reformation to enact the “defacing of the Libraries of their ancient records” (Speed, 1611, pp. 17–18; Summit, 2008, p. 3)? We know that certain books left Dominican convents due to lending and borrowing activity. This activity occurred frequently due to the order’s library philosophy, which discouraged friars from hoarding books or being unwilling to lend them (De Romanis, 1888/1889, pp. 418–432; Hinnebusch, 1973; Humphreys, 1995, pp. 132–133). Friars commonly inscribed entitlements in books to ensure against the loss of books that they entrusted to others (Hinnebusch, 1973, p. 204). We find palaeographical traces of borrowing in LJS 361: a fourteenth-century inscription on its inside back cover records that it was lent to the Dominican friar Umilis of Gubbio for a surety of one florin soon after it was written (Penn Libraries, n.d.).

It is probable that this friar never returned the borrowed book—books could be lent for long periods, even for life (Hinnebusch, 1973, p. 212). Alternatively, the scribe may have died, or passed the book to another lender after Umilis. It is also possible that the convent librarian sold the book: the influential Dominican Humbert of Romans encouraged librarians to sell duplicates and triplicates of texts—with the permission of the prior and on the understanding that the money would be reinvested in books (De Romanis, 1888/1889, pp. 418–32; Hinnebusch, 1973, p. 194; Kӓppeli, 1941 [1244], p. 10). Mandates against friars selling books to each other for more than they paid indicate that “trafficking” of books was a concern to legislators, and some books were even offered as security for loans (Hinnebusch, 1973, pp. 206–207).

We know that the book must have passed out of S. Domenico at some point in its early history since the other items in the convent’s library after 1861 were transferred to either the National Library or the University Library of Naples.^2^ Hinnebusch (1973) has observed that when the Order lost possession of a book, it was usual for the new owner to erase all marks of previous ownership (p. 219). The expurgation of the scribe’s name from the front flyleaf of LJS 361 indicates that a subsequent owner was eager to destroy a rival claim to ownership. Bale (2014) has described this type of purposeful erasure in his study of a fifteenth-century book owned by Dorothy Helbarton, MS HM 136. The book was “marked (or ‘damaged’) by Helbarton’s scribe in such ways as to efface a previous owner and to convert its value from an artefact for reading to an artefact for owning” (p. 91). The “silenced” voice (Bale, 2014, p. 97) of the scribe of LJS 361 was never superseded by later assertions of ownership. However, the erasure did convert the book “from one state to another” (Bale, 2014, p. 98). Having left the Dominican convent, this book was evidently taken into young hands and converted into its own new “state”. Before scrutinising the evidence for this encounter, I present a brief review of existing research into the relationship between pre-modern children and books.

## 3. Pre-modern books and children

How would this medieval book, surviving into the late-medieval period and beyond, come to be marked by children? To pre-modern book collectors, the users of manuscripts were the most dangerous—and least controllable—element of their long-term care. The abbot Johannes Trithemius in *De Laude Scriptorum* (1492–1494) expressed some confidence that subsequent owners of his books would treasure them: “why do we dwell on the care of books with many words? Those who love books doubtlessly treasure them and keep them even without a word from us” (As cited in Porck, 2011, p. 8). However, others were less optimistic about the long-term care of books, especially if they passed into the wrong hands. The author of *Hoemen alle boucken bewaren sal om eewelic te duerene* [*How one shall preserve all books to last eternally*], (1527), compiled a collection of rules on book “access, handling and storage”, aimed at ensuring that books lasted “many years …, yes, at least two hundred years” (Porck, 2011, p. 9).^3^ This text, probably aimed at children, indicates that the author had learned that these young people, themselves, were the book’s worst enemy. The last rule, added by the same scribe after the text’s completion, reads: “eighth, one should not let children learn from any books that one wants to preserve. Because whatever comes into their hands, as we see, it either stays there or it is ruined” (Porck, 2011, p. 9).^4^ Porck points out that this rule could have resulted from the “progressive insight” of the author: there was evidently a precedent for books being “ruined” (whatever that might mean) by children.

The fifteenth century can be regarded as the “age of libraries”, heralding “the consolidation of book collections into library rooms”—especially in religious and university contexts (Summit, 2008, p. 19). However, the survival of intriguing marks in medieval and early modern books, such as the chicken footprints across the open pages of a 1537 print of Tyndale’s Bible (Maclean, 2016; University of Glasgow, Sp Coll Bk8-e.11), testifies to the flexibility of early modern spaces for reading. So, with the feasibility of LJS 361 passing into young hands in mind, it remains to classify its marginal drawings as the work of children.

## 4. Children’s drawings in LJS 361: criteria for classification

**Figure 1.**
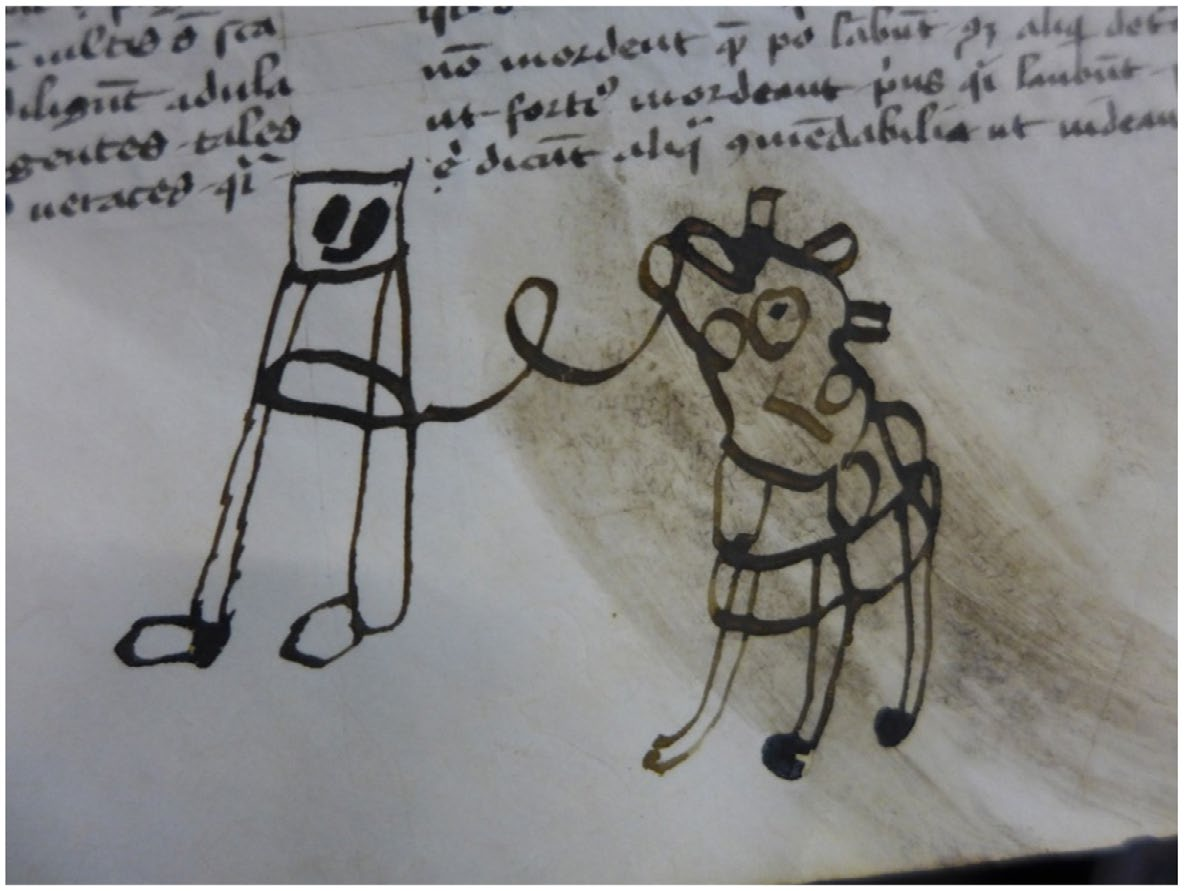
LJS 361, Kislak Center for Special Collections, Rare Books and Manuscripts, University of Pennsylvania Libraries folio 26r.

**Figure 2.**
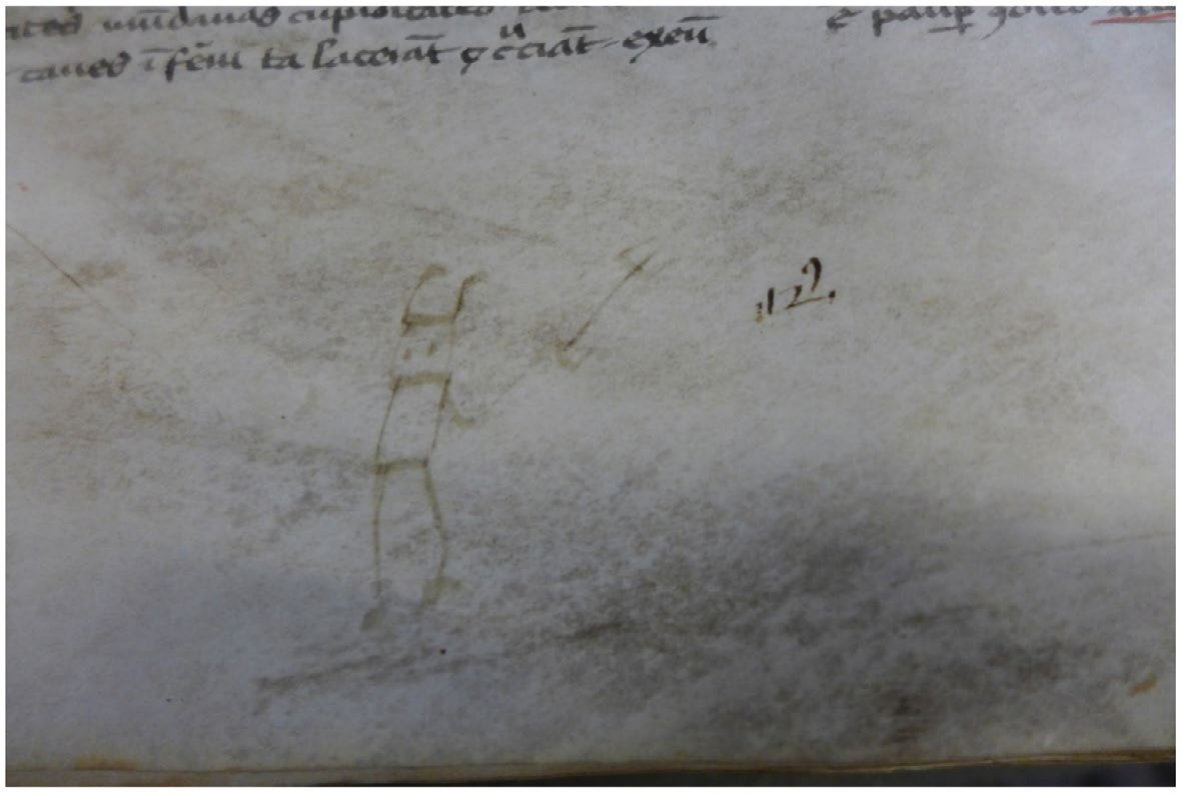
LJS 361, Kislak Center for Special Collections, Rare Books and Manuscripts, University of Pennsylvania Libraries folio 22r.

**Figure 3.**
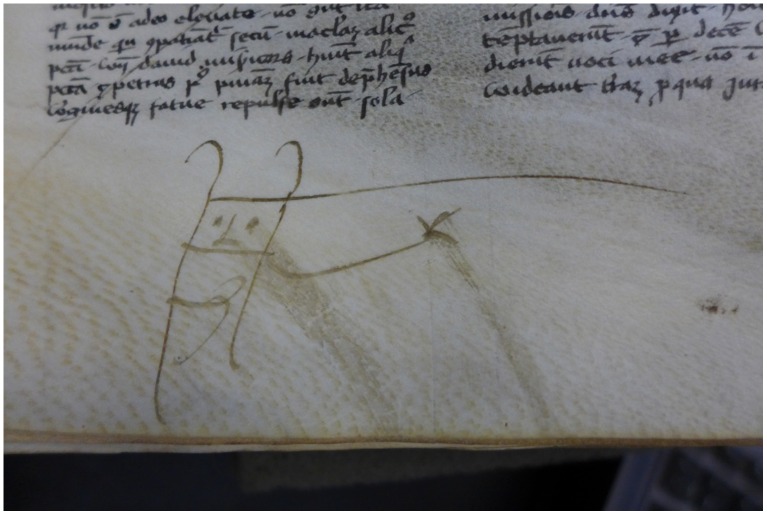
LJS 361, Kislak Center for Special Collections, Rare Books and Manuscripts, University of Pennsylvania Libraries folio 23r.

Three folios of LJS 361 have marginal drawings of human-like figures, along with one depiction of an animal—perhaps a horse or cow—which are included in the library catalogue under the category “early marginal drawings and notes” (Figures 1–3). This article argues that these doodles were the work of young children. It first acknowledges the general “child like” aspect of the drawings, before proceeding to delineate each feature that suggests the youth of the artists. This study draws from influential research in the field of developmental psychology, combining its principles with an examination of the material features of the drawings. This is followed by a study of doodles by adults in pre-modern manuscripts—drawings which, even at their most informal or crude, have stylistic features that separate them from the work of children.

### 4.1. General aspect

#### 4.1.1. They simply “look like” the work of children

To anyone familiar with the drawings of children, the images shown in Figures 1 to 3 give the impression of being the work of young hands. Why? Because, as Steel (2014) has pointed out in relation to an early modern drawing, they simply *look like* they are. Or, as Steel explains in more detail, because of a combination of features, including the “elongation of limbs” and the “enlargement of areas to accommodate detail … that can’t be rendered finely with a child’s typically gross motor skills”. Kwakkel (2013) has given further examples of medieval children’s doodles. In one, the child—apparently a schoolboy—sketches in his copy of a manuscript containing Juvenal’s *Satires*. In another, a thirteenth-century boy named Onfim doodles not in a book, but upon a scrap of birch bark found amongst miscellaneous Russian documents. These drawings are clearly all by children. Each one, as Steel (2014) argues in relation to the early modern drawing, “just *says* child”.

A tendency to refer to the general aspect of doodles—combined with a vivid imagination—has hitherto dominated studies of children’s drawings. For instance, Beard in her observations on the graffiti of Pompeii imagines: “the bored kids who scratched a series of stickmen at child height in the entranceway to a suite of baths, doodling as their waited maybe for their mothers to finish steaming” (2009, pp. 15–16). But what is it, specifically, about this “series of stickmen”—aside from their low positioning upon the wall—that indicates that they were made by children?

In an archaeological study of the Roman region of Campania, Huntley (2011) sets a precedent for the application of developmental psychology to historical drawings. Huntley’s work pushes beyond pertinent but imprecise statements such as “the drawing ‘just *says* child’” (Steel, 2014) to propose a systematic process for identifying children as the artists of ancient graffiti. Making reference to the findings of influential developmental psychologists, Huntley argues that it is possible to identify drawings as the work of children based on their stylistic features alone because “as a social group [children] are defined by physiological and psychological characteristics: their brains are developing and these changes, which in turn affect children’s capacity for visual representation, may be reflected in graffiti because the way in which children create such representations is directly related to their cognitive development (Efland, 2002; Huntley, 2011, p. 69; Kellogg and O’Dell, 1967; Sundberg and Ballinger, 1968). By applying her interdisciplinary approach, Huntley has identified 161 instances of children's graffiti in the sites of her study, with important implications for the study of children in the Roman world (2011, p. 69). Building upon Huntley’s initiative, I have devised a precise list of criteria for classifying drawings as the work of children, based on the findings of leading developmental psychologists. The following section analyses the doodles in LJS 361 in relation to these criteria.

### 4.2. Representation of human features

#### 4.2.1. The reduction of the human figure to the most important features

Psychologists have demonstrated that the earliest recognisable human figure drawn by children—after the initial scribbling phase of age around 12 months to 3 years—comprises what appears to be a head upon two legs, sometimes with facial features, known as the “tadpole figure” (Cox, 1993, p. 1). This figure reduces the human to its most important features, with a primary emphasis on the area most important to the child in their social interactions: the head and face. Two of these “tadpole figures” have been found in the ruins of Pompeii (Huntley, 2011, p. 74, Figure 4.1a).

**Figure 4.**
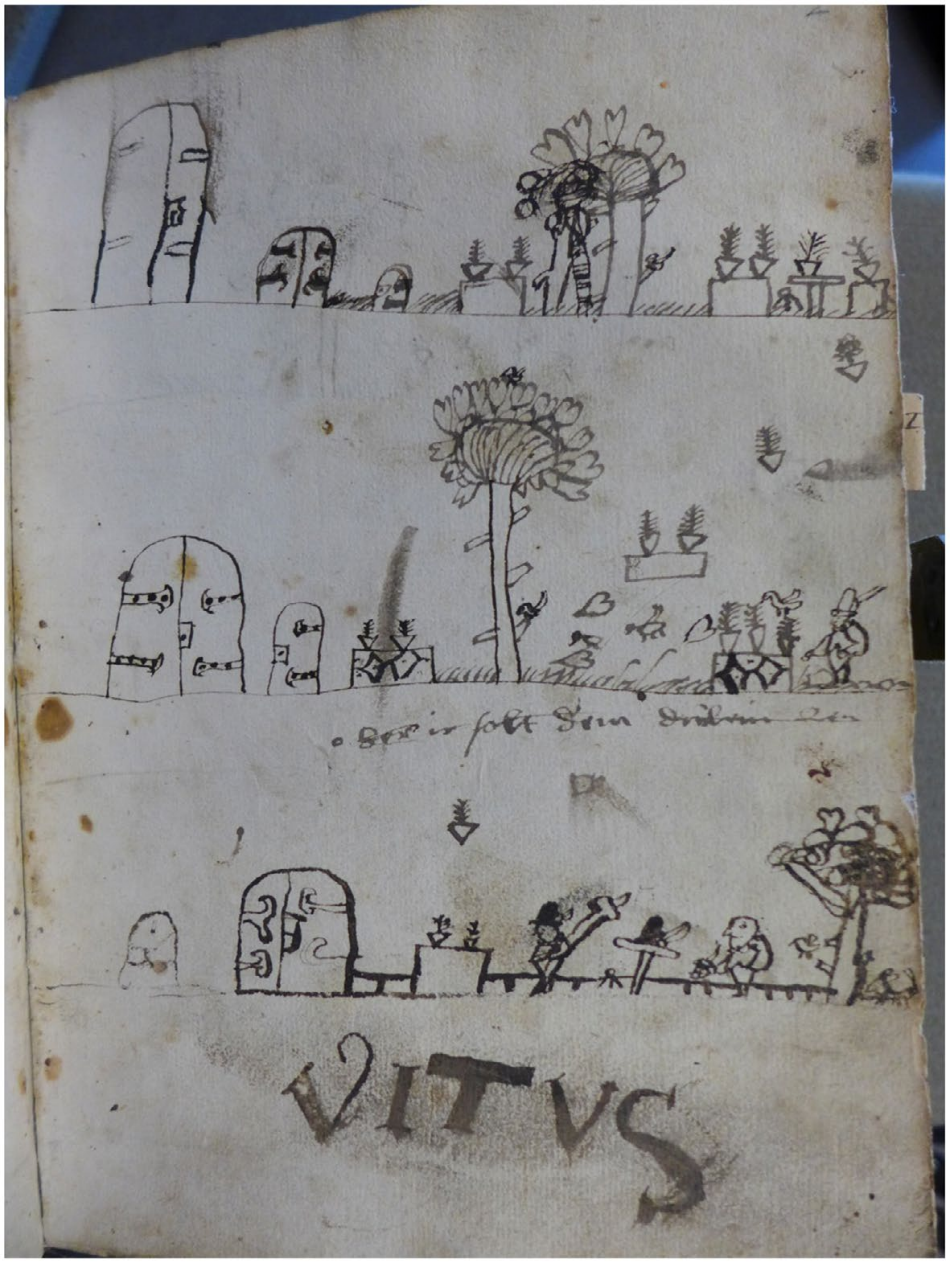
LJS 445, Kislak Center for Special Collections, Rare Books and Manuscripts, University of Pennsylvania Libraries folio 2v.

In personal correspondence (April 18, 2015), developmental psychologist Rosalind Arden of King’s College London indicated that the human standing to the left of the animal in Figure 1 is typical of this “tadpole” as drawn at around age 4.

Arden also observed that the human has filled-in eyes, consistent with the tendency to reduce features during this stage. As a result of the omission of the torso and arms, the animal’s lead rein is attached to the human’s legs.

#### 4.2.2. The formation of human features from geometrically regular shapes

The drawing shown in Figure 2 has the separate body component that is absent in the typical “tadpole figure” of very young children.

However, though this artist has drawn a structured figure comprising head, torso and legs, these components are rendered as unrealistic geometrically regular shapes. The figure has boxes for head and torso, as well as hooked “horns” protruding from the head and a three-pronged fork in the outstretched arm, indicating that it may be a devil. This simple figure is typical of young children: they identify a “salient shape” for each object to be drawn—e.g. the bulkiness of the head—and choose the most appropriate shape from his or her repertoire—e.g. a box—to correspond with his or her mental image of the object (Cox, 1993, pp. 14–18). This process is, as Goodnow terms it, a “search for equivalents”, and the scope for these equivalents increases with age (Arnheim, 1974; Cox, 1993, pp. 14–18; and 2005, pp. 59–61; Goodnow, 1977; Golomb, 1981; Willats, 1985, 1987). Each of the drawings in LJS 361, with the exception of the animal in Figure 1, comprises geometrically regular shapes (boxes) and lines. Huntley discusses a comparable graffito from Pompeii, in which the child artist combined geometrically regular shapes (a diagonal cross and an oval) to create a reuseable schema for the human figure (Huntley, 2011, p. 74, Figure 4.1a).

The denotation of both head and torso, and legs as boxes and single lines in Figure 2 indicates a young age, perhaps 4 to 6 years old, as the repertoire typically increases with age (though not universally, as is explained below). As the child develops, these regular shapes become more complex. For example, though the human in Figure 1 has legs emerging from its head, tadpole-style, they are “tubes”, which in most Western cultures today is typical of older children (Cox, 1993, pp. 17–18). Instead of using lines for limbs, Cox explains, the child moves towards making the figure’s body parts more realistic by creating “an outline of the shape of real legs” (Cox, 1993, p. 17).

#### 4.2.3. Economy in the use of different shapes

Once a child has chosen a shape from their relatively small repertoire, they typically use it repeatedly to represent different ideas—they demonstrate economy in their drawing (Cox, 1993, p. 49; Huntley, 2011, p. 75). Huntley (2011) observes this in her study of the Pompeii graffiti, noting that the same unit can be used to represent both arms and legs, as is the case in a graffito from the Casa dell’Criptoportico (p. 74, Figure 4.1b) This tendency is evident in Figure 2, where boxes are used to depict both head and body. Additionally, as in the Pompeii graffito, similar simple lines are used to denote both upper and lower limbs.

#### 4.2.4. A combination of shapes according to a schema

Young child artists tend to draw according to a schema, a preferred combination of shapes that are used and altered only slightly in denoting a variety of ideas. Cox (2005) observes this feature in a drawing in which a child of 3 years and 9 months uses the human “tadpole figure” as the basis of their drawing of a dog (p. 166, Figure 8.7).

This predisposition to pre-selected combinations of shapes offers clues about the identity of the child artist(s) in LJS 361. Figure 3 was evidently drawn according to the same schema as Figure 2: they are similar two-compartment figures with elongated arms and legs and prominent, curved, horn-like protrusions from the head. Both drawings were drawn with a crayon or pencil-like implement, as is discussed in more detail below. In contrast, Figure 1 human figure presents a different style, with a box-like head, big eyes and tubular legs, and appears to have been executed in ink. These differences between the adopted schema for these three drawings indicate that they were the work of at least two different children.

#### 4.2.5 Preference for “balanced” images

Children prefer “aesthetically well-balanced” images and often depict the different components of their subject projecting outwards (Kellogg, 1969, p. 34). Thus, the limbs of human figures often point out away from the central unit—the head or body—in a manner that is balanced rather than accurate, and suns and flowers are popular motifs for this reason (Huntley, 2011, p. 76; Kellogg, 1969, p. 34). The Casa dell’Criptoportico graffito from Pompeii is typical of this desire for balance, as its arms and legs are outstretched from the central unit of the head (Huntley, 2011, p. 74; Figure 4.1b). In LJS 361, Figures 2 and 3 illustrate this preference, with legs and one arm radiating from the central unit of the body, rather than in a more “relaxed” position.

### 4.3. Perspective and orientation

#### 4.3.1. The dominance of frontal/canonical orientation

Young children most often present the human figure in a frontal or “canonical” orientation (Cox, 1993, p. 5). The canonical orientation is the “object’s typical view and that which best displays its important structural or invariant features” (Cox, 2005, p. 73). Thus, a human figure would face the viewer, whereas a horse would be drawn side-on (Freeman, 1980; Gibson, 1979). The front-on depiction of human figures, like the dominance of the head discussed above, is due to the importance of face-to-face interaction for the socialisation of the child (Huntley, 2011, p. 75; Waksler, 1991, p. 13). This orientation can be seen in all three of the human figures in LJS 361 (Figures 1–3).

This contrasts with the side-on depiction of several human figures in the eighth- or ninth-century Inchmarnock “Hostage Stone” inscriptions, thought to have been made at the Scottish island’s monastery (see Lowe, 2007; pp. 53–68). Though these “cartoonish” inscribed figures have a childlike aspect, their orientation depicts the walking motion with more visual realism than is typical of a very young child. In addition, they do not reduce features as is conventional in young children’s drawings—rather, they have considerable detail in their attire and bodily features. They have, for example, cross hatching on the legs and, in the case of the “Viking” figure, a moustache and whiskers. Lowe has discussed the stone in relation to the practice of fostering children from the age of 7 within the monastery, and it is possible that this inscription was made by an older child (Lowe, 2008, p. 262). Similarly, the animal in Figure 1 is not depicted in its side-on canonical orientation, but instead is shown from the front. This indicates that it may have been contributed by an older child, who—like the artist of the Inchmarnock “hostage stone”—was able to explore more visually realistic ways of depicting their subject.

#### 4.3.2. Stiff poses

Human figures drawn by young children are notable for their stiff poses (Cox, 1993, p. 5). Huntley observed this rigidity in the graffiti from Pompeii, commenting that children “may draw a human figure reaching for something by adjusting the arms whilst the body remains upright, facing forward” (Huntley, 2011, p. 75). For example, in the graffito from the Casa dell’Criptoportico, the figure itself appears not to move, but its arms bend to reach something (Goodnow, 1977, p. 65; Huntley, 2011, p. 74 Figure 4.1b). The drawings in LJS 361 display similar rigidity, with the humans of Figures 2 and 3 in a static pose, with just one arm reaching out and slightly bent, and both human and animal in Figure 1 depicted standing still.

#### 4.3.3. Boundary preservation

Boundaries are important to the young child artist, so the different components of the human body rarely overlap. Huntley (2011) observes that children may add hair or ears to a circular head, but that these elements will not come in contact with the limbs (pp. 75–76). Cox (1992) points out that where children depict both head and trunk, these two areas will be represented by separate bounded regions, sharing a single boundary at the “neck” (p. 49). We see this feature in each of the drawings in LJS 361. The head and torso in Figures 2 and 3 are represented by separate shapes, which share one side at the “neck”. In Figure 1, the head and tubular legs are distinct areas. In contrast, the animal in Figure 1 has overlapping regions around the legs and back of the animal, again suggesting that a different, older, child may have been responsible.

#### 4.3.4. Intellectual realism, later transitioning into visual realism

Young children focus upon what they *know* rather than what they *see*, so their drawings will not necessarily depict the realistic visual features of their subject, but instead what they know *should* be there (Di Leo, 1970, p. 40; Huntley, 2011, p. 73). The child will draw a human front-on partly because this is the easiest way to include each of the features that they know are there (two eyes, a nose, a mouth, etc.). As the child grows older, they will move towards visual realism. Thus, they may depict a walking person side-on, or an animal front-on. This transition may not occur smoothly, as Huntley discovered in a graffito from Pompeii Grand Palaestra. This drawing shows a human figure turned to the side, but both arms are shown, and both eyes remain on the side of the face (Huntley, 2011, p. 75, Figure 4.1c).

The artist of the animal in Figure 1 has evidently passed through this transition; as Rosalind Arden has pointed out, the beast is drawn from a front-on perspective and its legs and other features are only those that would be visible from that viewpoint (personal communication, April 18, 2015; Cox, 1993, p. 5). This could be considered evidence for its production by an older hand, in line with the theory that children shift from representing what they know about an object (intellectual realism) to drawing what they can actually see (visual realism) with age (Cox, 2005, pp. 71–74; Luquet & Costall, 2001).

However, some caution should be exercised in using visual realism as an indicator of development. Cox (1993) has argued that visual realism itself has not been a universal feature of adult art over time, and that the drive towards this point-of-view realism is culturally driven (p. 5). For instance, her studies of the fourteenth-century Luttrell Psalter unveil a mixing of perspectives in art by adults that demonstrates a tendency towards intellectual realism rather than visual realism (Cox, 1993, pp. 168–169). This demonstrates that intellectual realism is not a strictly “childish” convention (Cox, 2005, p. 87). In addition, though evidence indicates a general movement towards visual realism around the age of 7 or 8 years old, Cox (2005) has shown that visual realism is possible in younger children and, equally, that the habits of intellectual realism may continue in older children and even adults (p. 74, p. 88). With this in mind, Huntley refuses to assign ages to the child artists of Roman graffiti, arguing that it is difficult to know the rate of their cognitive development in relation to modern children. Citing modern studies demonstrating that children who are taught to draw show faster development (Alland, 1983, p. 203), she chose instead to assign all of the ancient graffiti to a single category (“below the age of 12”) (Huntley, 2011, p.78).

Regardless of these arguments against rigid stage-like “shifts” in perception abilities, this collection of drawings demonstrates varying degrees of visual realism. The representation of the animal in Figure 1 contrasts markedly with the human figure next to it. Its portrayal from a front-on perspective is consistent with an older child’s search for more realistic ways of representing things (Cox, 2005, pp. 90–97, p.177; Golomb, 1981; Goodnow, 1977; Luquet & Costall, 2001). There is a clear difference in perspective between the two elements of the drawing shown in Figure 1, and this coincides with other features that suggest that the animal was contributed by an older child.

### 4.4. Size

#### 4.4.1. Large head

Children often draw the human head too large for the torso (Cox, 1993, p. 62). The most convincing explanation for the oversized head is that it is due to the child’s still-developing planning skills (Thomas and Tsalimi, 1988). As Steel (2014) suggests in relation to the drawing in Melusine, a human head includes many details, and the child anticipates having to fit them all in by exaggerating its size (Cox, 1993, p. 63; Freeman, 1980). Additionally, the head is often drawn first, so it gets “first choice” of the allocated space (Cox, 1993, p. 62). However, inspecting the human heads of Figures 1–3, we see that though the head of Figure 2 is much too large for its body, the humans in Figures 1 and 2 have reasonably sized heads.

#### 4.4.2. Elongated limbs

As Steel also observes in the Melusine doodles, drawings of humans by children often feature elongated limbs. Cox explains that children’s drawings are generally taller than they are wide, which reflects, but exaggerates, the proportions of real people (Cox, 1993, p. 62). Figure 1 demonstrates this tendency clearly, as its long legs are almost twice the length of the adjacent animal figure. Figure 2 outstretched arm is longer than its legs, giving it highly unrealistic proportions. With experience, children develop the ability to better portray the true height–width ratio of human figures (Cox, 1993, p. 62; Schuyten, 1904).

### 4.5. The material evidence: stylus and inking

It remains to scrutinise the material evidence, gathered during an examination of the manuscript in person, which supports my assertion that the drawings in LJS 361 were the work of children.

#### 4.5.1. Writing implement

The main text of the central section of this manuscript (folios 10r−42r), in which all of these doodles appear, is written in a single small, neat, fourteenth-century hand. The margins of the text contain some marginal annotations and decorated catchwords, which are of the same colour and ink consistency as the main text and so are almost certainly in the same hand. In contrast, qualities of ink colour, thickness and consistency in all three of the drawings set them apart from the main text. This observation, whilst not in itself proof of the youth of the artists, demonstrates that the drawings were not part of the manuscript’s programme of design. Figure 1, like the main text, was executed using a quill, but a thicker one than was used for the main text. The ink of this figure is notably darker and thicker than that of the main text and any other decoration in the book (such as the decorated catchwords on folios 21v and 33v). Figures 2 and 3 appear to have been created using a brownish waxy crayon-like implement, most similar to that used to rule frames at certain points in the book. The similarity in the writing implement used to make these two drawings supports the stylistic evidence that they are by the same artist.

#### 4.5.2. Stylus control

A child typically shows imprecision in pen control compared to even the most unskilled adult, reflecting their developing motor abilities. Looking at the human in Figure 1, Arden has suggested that the thickness of the line has not been regulated using the nib, implying that the child has not developed the angled grasp necessary to produce an elegant line (personal communication, April 18, 2015).

Close examination of the individual quill strokes reveals that the straight lines of the legs are jagged, suggesting a slow, unconfident, hand movement, rather than the practiced glide of a more developed hand (Figure 1). In contrast, the drawing of the rein/lead of the animal appears to be executed by a more skilful hand, which creates a smooth line with variations in thickness that curve elegantly in the middle (Arden, personal communication, April 18, 2015). Inspection of the manuscript reveals that the lines of the animal are on average slightly thicker than those of the human. There is lighter inking in this region—most noticeable around the eye, mouth and nose of the creature. This observation gives the impression that these two parts were drawn by different artists. The crude motor control evident in even the most accomplished parts of this drawing contrasts with the decorated catchwords in LJS 361. The elegant catchwords comprise finely detailed boxes and zig-zag lines, in one case interspersed by lines executed in the red pen otherwise used for rubrication in this manuscript (see folio 21v).

#### 4.5.3. Smudging

Smudging is a dominant feature in both Figures 1 and 3. In Figure 1, the animal has a smudge passing through it, which does not impinge on the adjacent human. The lines of the animal and its lead rein are smooth, suggesting that the smudging is either underneath this figure, or it was made with fresh ink after the drawing had dried. If the former was the case, it could indicate that an earlier attempt was erased. This supports the argument—first suggested to me by Rosalind Arden—that the human figure was drawn by one child, with the animal and its lead rein contributed by another. This smudge also helps us date the doodles to after the folios were bound into a book format, as it has left an imprint on the facing folio, suggesting that the book was closed before the ink was dried.

The smudging in Figure 3 is localised, extending only from the hand and face of the figure. Both of these small smudges are directed downwards and slightly to the right, suggesting that they were made with the right hand whilst the child was drawing.

Smudging is also seen in a flyleaf drawing in LJS 445, a manuscript copy of astrological predictions from around 1,500, extending upwards from the door in the top left of Figure 4.

This image exudes childishness in its repetition of schemas (for example, in the doors, trees and birds); its “lollipop” trees with stylised heart-shaped leaves; and its clumsy lines with little regulation of thickness. However, it also displays side-on (rather than canonical) human figures wearing hats, ornate collars and pantaloons. The drawing conveys motion, as leaves fall, birds fly and people walk. These figures witness the slow replacement of intellectual realism with visual realism as a child ages, as well as the increasing repertoire of dynamic postures of the human figure, moving beyond the static, canonical, depictions typical of younger artists.

### 4.6. Date and geography

There are no features in the drawings in LJS 361 (items of clothing, hairstyles, buildings and/or inscriptions, for example) that help date them. Perhaps one cultural issue to be noted is the preference for rectangular shapes in the drawings of modern-day children from Africa and the Middle East (Cox, 2005, p. 222). However, though Figures 1 and 2 display rectangular torsos consistent with what Wilson and Wilson (1984) term the “Islamic” torso, there is no evidence to link the drawings to a particular geographical region. Developmental psychologist Esther Burkitt has pointed out that the shape of the heads seen here is very rare in drawings made by children today (private correspondence, May 2015). As is explained below (pp. 13–14), there is a wealth of evidence for physical encounters between medieval books and early modern children, which may help date the drawings to some time in that period.

## 5. Doodles by adults in pre-modern books

It should be recognised that the leaves of manuscripts were not only vulnerable to the hands of children. In fact, the most prolific doodlers in medieval books were adults. Thus, this section proceeds to consider some doodles by adults, giving more attention to their playful aspect, whilst delineating the features that separate them from drawings by children. Interest in marginal illustrations in medieval manuscripts grew in the mid-to-late twentieth century, as scholars recognised that the margins of medieval books should not be overlooked in a process of analysing the text, but should be examined as part of the book as a whole. Michael Camille’s seminal *Image on the Edge* (1992) demonstrated that marginal illuminations were not always decoration to the main text, but should be considered a secondary text, interacting with and commenting with, its contents (pp. 11–12). Pulsiano (2002) has added that these illustrations could have a range of functions: “sometimes ornamenting, sometimes competing, sometimes commenting on the text they surround” (p. 198).

However, as Pulsiano shows, scholarly attention has focussed on the “more rich and entertaining margins”—particularly those whose absurdity appeals to our modern sensibilities (for example, “[a] monkey-like creature mounted on an ostrich”, 2002, p. 189). In contrast, pen doodles—neither part of the text nor an elaborate scheme of decoration—can slip through the cracks of codicological scholarship. This is despite the fact that many readers made connections between space and text that offer insight into the transmission and use of medieval texts.

Not all marginal drawings by adults display artistic flair, obvious meaning or sophistication, though, which has contributed to their neglect. Surveying marginal doodles made in Anglo-Saxon manuscripts, Pulsiano (2002) declares some of them “elegant and suggestive in their simplicity … offering Picasso-esque representations of the human form” (p. 190). The drawings he examines include a human figure constructed from boxes, with the written statement in his torso: “this is man” (Pulsiano, 2002, p. 190). There is what appears to be a chicken–human hybrid grotesque and what Pulsiano describes a “melon-headed figure with bulbous eyes” (2002, Figure 2; p. 190). However, rather than being the work of playful children, the doodles are signs of adult readers and scribes at play: “such doodles bring us into the world of modest play, of readers and scribes seeking distraction” (Pulsiano, 2002, p. 190). They represent an “urge to interrupt the silence of blank page” (Pulsiano, 2002, p. 190). What is it about these drawings, with their simplistic qualities, bearing no relation to the text they surround (Pulsiano, 2002, p. 190), that indicates that they are the work of adults and not children?

Pulsiano himself was confounded by doodles as material records of human interaction with the material text, but devoid of further contextual clues: “we will never understand in nearly all cases why a head is tossed into the margins here, a chicken there, or what impelled these users to leave their anonymous marks” (2002, p. 195). However, he urges codicologists to take note of them as witnesses of “playful activity and creative urges at work” (Pulsiano, 2002, p. 195). Though playful, the drawings he studies have qualities that suggest they were made by adults, or older children, rather than young children. The lines are smooth and deliberate, despite their abstract “Picasso-like” nature. Drawings of faces have all of the constituent features: eyes, noses, eyebrows and mouths. Some have detailed hats with decorative adornments, and others have collars and hair made up of wavy lines indicating curls. Heads are rounded or realistically shaped, often culminating with chins, and given ears, which contrasts with the reduction in features typical of drawings by children. Despite their absurdities, these drawings just look like they were contributed by adults.

**Figure 5.**
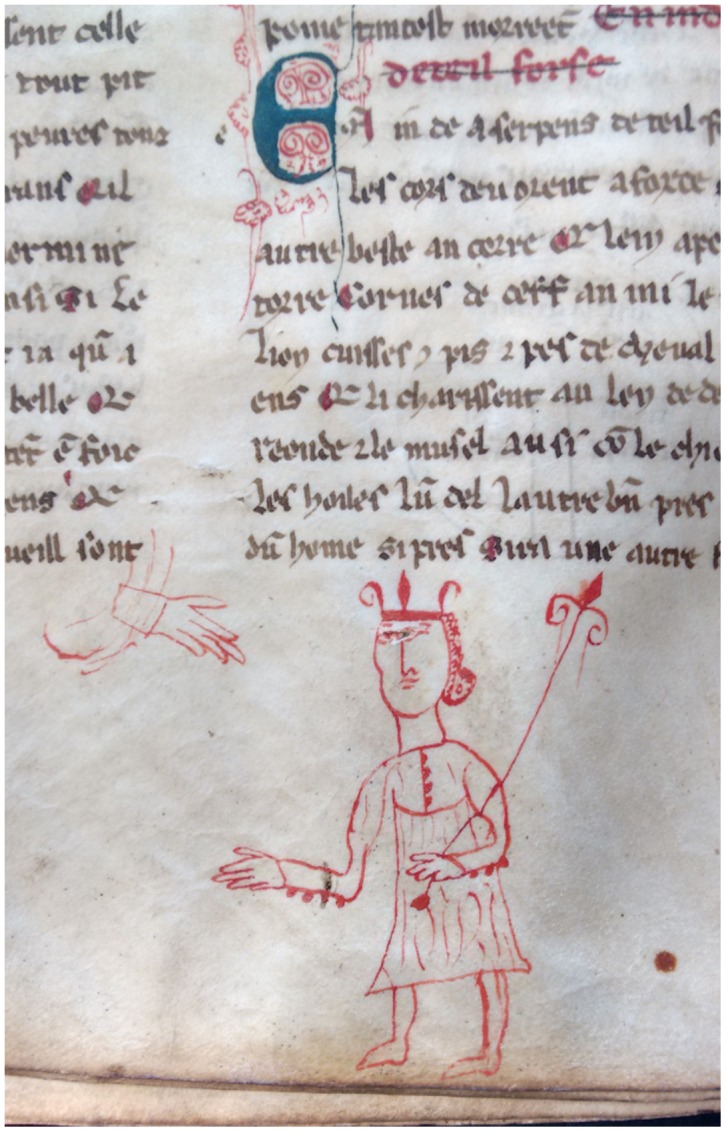
LJS 55, Kislak Center for Special Collections, Rare Books and Manuscripts, University of Pennsylvania Libraries folio 10v.

Medieval books abound with doodles that, despite their playfulness, are likely to be the work of grown hands. For example, in Figure 5, a thirteenth-century copy of Gautier’s *L*'*Image du Monde*, there is a marginal drawing of a king being blessed by the hand of God. This king appears to have been drawn using the same red ink as the folio’s decorative flourishes. The figure also shares stylistic features with the book’s decorated initials. For example, his hair comprises a similar curly pattern to the flourish around the letter “E” above him. These features indicate that this drawing was part of the decorative programme of the book, despite its naïve appearance.

To further scrutinise this doodle, the king’s gesticulating arm is comically out of proportion with the rest of his body, his eyes are scrunched together in his forehead, his nose is depicted side-on, despite his canonical orientation, and his hand does not grasp his sceptre, but is instead drawn with its fingers extended. However, regardless of this lack of sophistication, the king’s stylistic features indicate that he was the work of an adult hand. There is accuracy in pen control, as the artist creates contrasts between thin lines and in-filled areas such as his crown and hair. There is attention to detail and proportion in the king’s facial features, hands and fingers, and in his paraphernalia. The human figure is one fluid shape comprising head, neck, clothed body, arms and legs—which contrasts with the box-like components in Figures 2 and 3. The fact that this figure has a neck at all is an indication that this is the work of older hands: young children rarely give necks to their figures (Cox, 1993, p. 62; cf. Figures 1−3). Finally, the artist has paid attention to the king’s elaborate clothing, detailed down to its buttoned sleeves and textured tunic. He has elegantly pointed shoes, which contrast with Figure 1 rounded stumps and the lack of feet in Figure 3. This comparison demonstrates that despite the crude appearance of some marginal drawings by adults, they can be distinguished from the work of children by features that reflect their advanced level of cognitive development.

## 6. Codicological implications

Lerer (2012) has explained the irresistibility of a book’s margins to children. In introducing his own research into children’s marginalia, he refers to Hunt’s declaration (1890) that, to the child, “the margin is the best part of all books, and he finds in it the soothing influence of a clear sky in a landscape” (p. 126; Hunt, 1890, p. 85). Hunt traced the child’s inclination to make a mark from his “first impulse” to scribble on the wall or a fresh sheet of paper, through to a later desire to write and draw *around* the text, in the margins of school books (1890, p. 85). Lerer also provides Kenneth Grahame’s poetic view of these marks, describing “crocodiles and monsters” in scholarly texts, “amorous missives” in hymn books and “superior rhymes” written in the margins of printed books (Grahame, 1894; Lerer, 2012, p. 126).

Though Lerer’s research ranges from “infantile unlettered marks” to “carefully scripted signatures”, its focus is on the annotations of older children—who would today be school age—in medieval books. The children of LJS 361 were neither infants nor older children, so sat somewhere in the middle of Lerer’s range. Their doodles witness interactions between at least two young children and a medieval book. This section examines the codicological context of these three doodles, considering the implications for our knowledge of the lives of medieval material texts. It explains that the drawings may bear some relationship with the content of the text, which might suggest that the children had some understanding of its subject matter.

Bale (2014) argues that we should resist the temptation to use marginal inscriptions in manuscript books “as supporting and secondary evidence” (p. 92). Instead, he argues that “a book’s marks, its damage, and its paratexts can be more illuminating, culturally, than the so-called main body and text” (Bale, 2014, p. 92). This is true for LJS 361; its drawings form a disjoint with its “so called main body”. Whilst this “main body” is a specialised compilation of texts produced within the institutional context of a Dominican convent in Naples, the drawings capture the playful activities of young children. The book contains little other evidence of its use after its fourteenth-century inscription, which should have recorded a fleeting passage into the hands of another Dominican friar before it was returned to its rightful owner.

If the marks in LJS 361 were made by children, as the stylistic and palaeographical evidence suggests, they are evidence for medieval books being stored and read in the vicinity of children. This has already been observed by Lerer, who shows that whilst copies of the Chaucer’s *Canterbury Tales* were popular in the sixteenth century, some copies were neglected by their owners, and children often played in parental libraries (2012, p. 131). He finds evidence in the writings of playful older children: for example, in the fifteenth-century Helmingham Manuscript (Princeton University Library MS 100) containing an almost complete copy of the *Canterbury Tales*, there is the inscription “Alsabatha carman haue rent a pas a paper” (“Elizabeth Carman has ripped a piece of paper”) at the bottom of the “Tale of Melibee” in childish scrawl (Lerer, 2012, p. 131). Evidently, Carman’s childish exuberance resulted in her mistakenly, or purposefully, ripping some paper (though not within this manuscript itself), which someone felt the need to signal in writing in this book. LJS 361 contains evidence for encounters between children and medieval books; one or more children used its folios to test their developing repertoire for pictorial representation. They may have been laying the foundations for an eventual ability to write: drawing as young children helps us develop the fine motor skills that we use to execute letters (Arden, Trzaskowski, Garfield, & Plomin, 2014; Saida & Miyashita, 1979). Furthermore, as I explain below, the drawings may have a symbolic relationship with the “main body” of the text, suggesting some literate relationship between child and text.

**Figure 6.**
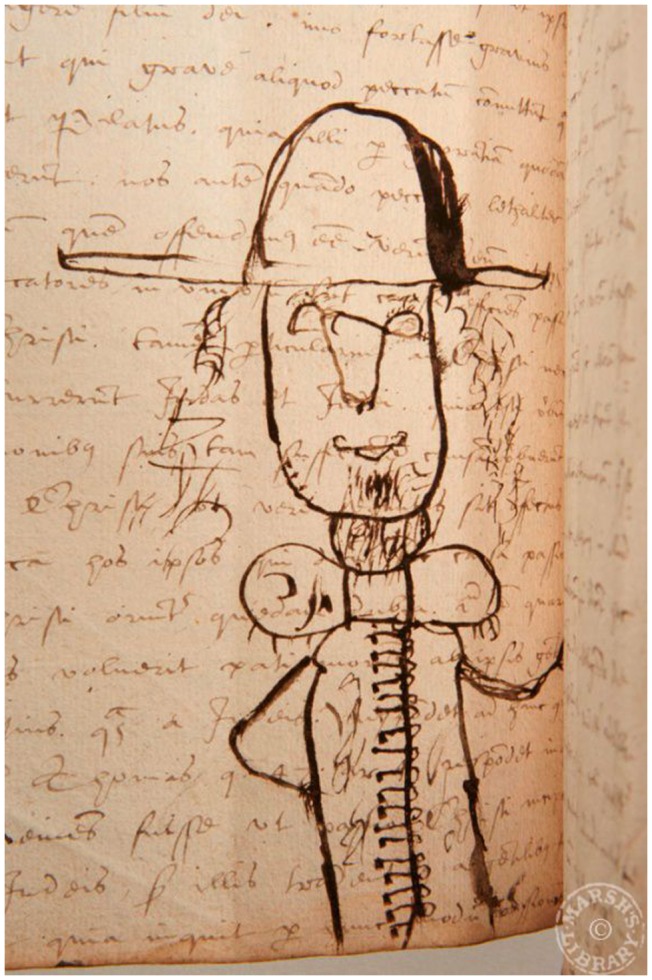
Dublin, Archbishop Marsh’s Library, Z4.4.7. Doodle in a sixteenth or seventeenth-century manuscript volume entitled Disputationes Theologicae.

Source: Marsh's Library©, Dublin

A sophisticated relationship between child and text is indicated by the young artists’ avoidance of the text of LJS 361. Instead of defacing the text, they restricted their drawings to the margins, to the extent of squeezing the human head into the gap between two columns of text (Figure 1). They, like the school children described by Hunt (1890), drew “*around* the text” (1894). Compare this reverence for the text with the human figure depicted in Figure 6.

This ambiguous drawing is childish in its general aspect, its evidence of poor pen control, and exaggerated size, but adult like in some of its features. Unlike the drawings in LJS 361, the artist has provided a significant amount of detail, with buttons on the coat, a beard and flowing hair, and what appear to be eyeglasses. Unlike the conventional figure by a young child, this human figure has a neck, and arms in a dynamic pose, as if gesticulating to the reader. This page also contains an abortive, enlarged, attempt at writing a sentence, by an unpractised hand similar to the writing in the child’s primer studied by Acker (2003, p. 145). Whether this human figure was drawn by an older child or an unpractised adult, the artist clearly saw little value in the book’s contents: he or she obliterated the text unapologetically.

In contrast, the features of the drawings in LJS 361 suggest that the artists were children who understood what *text* was and left it untouched. This is consistent with Bottigheimer's observation in relation to medieval Bibles: that children “scribbled on the endpapers and title pages but generally treated the text as inviolably sacral space” (1996, p. 6; Lerer, 2012, p. 130).^5^ There is some evidence that the child artists of LJS 361 may have some understanding of the text itself. There may be a relationship between the contents of the text and the subject matter of the doodles. Transcriptions and translations provided by Jessica Lamothe reveal that the text at the foot of the first column of folio 26r (Figure 1) from the sermons of Durandus concerns “false flatterers” who gain the pleasure of prelates, whilst men of truth are “held abominable” (personal communication, April 21, 2016). The text employs the metaphor of a scabby horse (*equus scabiosus*) that allows itself to be gently anointed (*leniter ungatur*) but not groomed (*strillietur*). In this analogy, the liar anoints (with flattery) whilst the truthful man is he who grooms and lances/heals (*strilliat et pungit*). The drawing of the man leading an animal, possibly a horse, may connect with this part of the text (Figure 1).

Lamothe has shown that the devils drawn on folios 22r and 23r (Figures 2 and 3) may also relate to the text, which has brief references to the torments of devils (personal communication, April 21, 2016). For instance, in the second column of folio 22r, Durandus’ text employs the metaphor of a stag, seeing itself surrounded by dogs, weeping and escaping to revive itself at a spring. The text refers to Psalm 22:16, “many dogs have surrounded me”, explaining that the dogs represent demons. If these drawings have some symbolic relationship to the text, we must ask: What are the implications for our understanding of pre-modern child education and literacy?

## 7. Conclusion

In the planning stages of this article, developmental psychologists Rosalind Arden and Esther Burkitt inspected the drawings of LJS 361, and judged them to be the work of children. Arden commented that the human in Figure 1 is of the “tadpole” type figure typical of a four-year old, whilst the animal shows signs of being slightly older (personal communication, April 18, 2015). Burkitt placed the age of the child artist of Figures 2 and 3 at approximately five years old (personal communication, May 6, 2015). By developing a list of criteria, based on the stylistic features of modern drawings by children, I can argue with confidence that the drawings in LJS 361 were the work of children.

Close scrutiny of the material features of these drawings in person supports this assertion, and indicates that that there was more than one child artist involved. For example, the hesitant, jagged lines of the human in Figure 1 contrast with the smooth strokes in the adjacent animal, suggesting different artists. In addition, there are minor differences in the ink colour and consistency between these two regions of the drawing. Finally, there is smudging around the animal, which may suggest that an original attempt was erased.

In a recent exhibition, children’s marginalia was exhibited alongside page rips by dogs and even rat droppings caught within the volume—each various and striking “defacements” of the book (Lerer, 2012, p. 128). Lerer argues that the pen work of children should not be considered defacement, and the doodles in LJS 361 support this argument. The children responsible doodled in this medieval book gleefully, but they restricted their drawings to the margins, and may have even had some understanding of the subject matter of the text itself.

The effacement of the original scribe’s name from the first folio of LJS 361 hinted that one early possessor wished to convert the book into their “artefact for owning” (Bale, 2014, p. 91). However, without these drawings, the cultural context of the texts within the manuscript, and the provenance of the manuscript itself, might appear unremarkable. Its subsequent owners would have otherwise been lost to history, along with the many other individuals who have looked upon medieval folios but not left a mark. Instead, the crude but appealing images that survive in LJS 361 deepen both our understanding of the use and reuse of medieval books, and our knowledge of human development in historical context.

This study suggests that young children were allowed access to this fourteenth-century book. If the doodles in LJS 361 do bear a symbolic relationship with the text, did the children use this medieval book in the process of developing literacy, or was it read to them by others? These are future research questions relating to the education of pre-modern children, and the role that of medieval books in that process. This study widens the field of pre-modern codicology by providing material evidence that young children were part of the life of medieval books. It offers an analytical method for separating the drawings of children from childlike drawings by adults, based on the most authoritative works in developmental psychology. Altogether, it presents drawings that are an endearing record of the intellectual development of pre-modern children as they learned, interacted and played.

## Notes

1. For an introduction to debates around the function of medieval images in the margins of books, see Camille, 1992.

2. The author would like to thank Mario Gaglione for this information.

3. “books will last ‘menich jaer […], ja te minsten twee hondert jaer” (p. 9).

4. “Ten 8sten, men sal huut gheenen boucken diemen ter heeren hauwen wilt, de kinderen laten leeren. Want wat in haerlieder handen comt, soe wij sien het blijfter oft het bedeerft”.

5. Lerer mis-cites “Rosemary Bodenheimer”.
